# Potential source of bias in AI models: lactate measurement in the ICU in sepsis patients as a template

**DOI:** 10.3389/fmed.2025.1606254

**Published:** 2025-07-09

**Authors:** Pratiksha Pradhan, Fredrik Willumsen Haug, Nebal S. Abu Hussein, Dana Moukheiber, Lama Moukheiber, Mira Moukheiber, Sulaiman Moukheiber, Luca Leon Weishaupt, Jacob G. Ellen, Helen D'Couto, Ishan C. Williams, Leo Anthony Celi, Joao Matos, Tristan Struja

**Affiliations:** ^1^Laboratory for Computational Physiology, Institute for Medical Engineering and Science, Massachusetts Institute of Technology, Cambridge, MA, United States; ^2^College of Engineering, Northeastern University, Boston, MA, United States; ^3^Harvard John A. Paulson School of Engineering and Applied Sciences, Boston, MA, United States; ^4^Pulmonary Critical Care Sleep Medicine Division, Yale School of Medicine, New Haven, CT, United States; ^5^Picower Institute for Learning and Memory, Massachusetts Institute of Technology, Cambridge, MA, United States; ^6^Department of Computer Science, Worcester Polytechnic Institute Computer Science, Worcester, MA, United States; ^7^Harvard Medical School, Boston, MA, United States; ^8^Division of Pulmonary, Critical Care, and Sleep Medicine, Georgetown University Hospital, Washington, DC, United States; ^9^School of Nursing, University of Virginia, Charlottesville, VA, United States; ^10^Department of Biostatistics, Harvard T.H. Chan School of Public Health, Boston, MA, United States; ^11^Division of Pulmonary, Critical Care and Sleep Medicine, Beth Israel Deaconess Medical Center, Boston, MA, United States; ^12^Faculty of Engineering, University of Porto (FEUP), Porto, Portugal; ^13^Institute for Systems and Computer Engineering, Technology and Science (INESCTEC), Porto, Portugal; ^14^Medical University Clinic, Kantonsspital Aarau, Aarau, Switzerland; ^15^Hospital Muri, Muri Aargau, Switzerland

**Keywords:** sepsis, lactate, MIMIC-IV, critical care, health equity

## Abstract

**Objective:**

Health inequities may be driven by demographics such as sex, language proficiency, and race-ethnicity. These disparities may manifest through likelihood of testing, which in turn can bias artificial intelligence models. We aimed to evaluate variation in serum lactate measurements in the intensive care unit (ICU) in sepsis.

**Methods:**

Utilizing MIMIC-IV (2008–2019), we identified adults fulfilling sepsis-3 criteria. Exclusion criteria were ICU stay < 1-day, unknown race-ethnicity, < 18 years of age, and recurrent ICU-stays. Employing targeted maximum likelihood estimation analysis, we assessed the likelihood of a lactate measurement on day 1. For patients with a measurement on day 1, we evaluated the predictors of subsequent readings.

**Results:**

We studied 15,601 patients (19.5% racial-ethnic minority, 42.4% female, and 10.0% limited English proficiency). After adjusting for confounders, Black patients had a slightly higher likelihood of receiving a lactate measurement on day 1 [odds ratio 1.19, 95% confidence interval (CI) 1.06–1.34], but not the other minority groups. Subsequent frequency was similar across race-ethnicities, but women had a lower incidence rate ratio (IRR) 0.94 (95% CI 0.90–0.98). Patients with elective admission and private insurance also had a higher frequency of repeated serum lactate measurements (IRR 1.70, 95% CI 1.61–1.81 and 1.07, 95% CI, 1.02–1.12, respectively).

**Conclusion:**

We found no disparities in the likelihood of a lactate measurement among patients with sepsis across demographics, except for a small increase for Black patients, and a reduced frequency for women. Subsequent analyses should account for the variation in biomarker monitoring being present in MIMIC-IV.

## Introduction

Disparities in healthcare are widely recognized, especially regarding discrimination based on race and ethnicity (1, 2). Such disparities can unveil themselves as differences in quality of care, unequal medical device performance, or access to services reflecting structural inequities (3). These biases are not only harmful for patient care, but can also impact the development of machine learning-based clinical algorithms that train on electronic health records (EHR) (4).

Ensuring the development of fair AI models is crucial, and addressing missing information is a key initial step in achieving this objective, especially when such information is not missing at random (5, 6). Unfortunately, this variation in the level of monitoring is often not taken into consideration in the development of machine learning-based clinical algorithms. In a 2017 study that evaluated 107 electronic health record (EHR)-based risk prediction tools, 49 did not account for missing data (7). A common approach to imputation is the use of normal values based on the assumption that laboratory tests that are not ordered are presumed to be within normal range, a practice that likely introduces bias (8).

The probability of detecting an abnormal finding is contingent on the frequency of testing. Consequently, non-randomly missing data can lead to spurious correlations—non-causal relationships between features and outcome—that are learned and then incorporated into clinical algorithms (9). When the etiology of missing data stems from social determinants of care, these biases can become ingrained in subsequent AI models, perpetuating, and even scaling existing disparities (10, 11). This is even more important in a high-stake environment such as in patients with sepsis admitted to the intensive care unit (ICU).

Sepsis is a severe life-threatening systemic infection and effective management of this condition requires prompt diagnosis, aggressive treatment, and continuous monitoring. Despite current advances, one key challenge remains the timely delivery of care. Herein, serum lactate level is one of the two key diagnostic tools of septic shock according to the guidelines (12, 13). Disparities in sepsis outcomes are known to exist (14). However, the drivers of sepsis disparities are unknown and the question of whether disparities extend to serum lactate monitoring remains underexplored.

This paper seeks associations between race and ethnicity, sex, and language and the frequency of serum lactate determination during the management of sepsis in the ICU. By shedding light on this dimension of care, we aim to contribute to a more comprehensive understanding of the social patterning of the data generation process in healthcare.

## Methods

This observational retrospective study is reported in accordance with the Strengthening the Reporting of Observational Studies in Epidemiology (STROBE) statement (15). The health equity language, narrative, and concepts of this paper follows the American Medical Association's recommendations (16).

### Data extraction

Data was extracted from the publicly available MIMIC-IV database (17). The MIMIC database is maintained by the Laboratory for Computational Physiology at the Massachusetts Institute of Technology and shared via the PhysioNet platform (18). The dataset has been de-identified, and the institutional review boards of the Massachusetts Institute of Technology (No. 0403000206) and Beth Israel Deaconess Medical Center (2001-P-001699/14) both approved the use of the database for research. The MIMIC-IV database includes physiologic data collected from bedside monitors, laboratory test results, medications, medical images, and clinical progress notes captured in the electronic health record from patients admitted to the ICU between 2008 and 2019.

### Hypothesis

We hypothesized that both the likelihood for a patient to have a serum lactate measurement and the frequency of subsequent measurements are not the same across race-ethnicity, sex, and English proficiency (as recorded by providers).

### Cohort selection

The following exclusion criteria were applied to create a study cohort: those without sepsis as defined by the sepsis-3 criteria (12), patients under 18 years of age, and those with length of ICU stay [length of stay (LOS)] <1 day. Patients with recurrent ICU-stays in the database, and those with a racial description other than White, Asian, Black, or Hispanic, especially excluding those of the heterogenous group “other.” For the negative binomial regression, we further excluded patients with absent serum lactate values on day 1.

### Covariates

We drew directed acyclic graphs (DAG) to understand which variables to extract (Supplementary Figure S1, Supplementary Table S1). Twelve confounders were extracted, including non-time-varying variables such as demographics, comorbidities, admission information, and source of infection and time-varying variables including Sequential Organ Failure Assessment (SOFA) score (19), and fluids normalized by LOS. Time-varying variables were modeled as follows: SOFA score was calculated for the day of ICU admission; serum lactate measurements were used as a binary variable for whether or not it was measured on day 1, in addition to taking the overall number of measurements for the whole ICU stay normalized by LOS.

### Outcomes

We had two primary outcomes: the first was a binary variable predicting whether a patient received serum lactate measurement on day 1; the second was the number lactate measurements a patient would receive divided by the number of days in the ICU (LOS).

### Statistical analysis

Statistical analysis was performed using Python 3.10.9 (20) and R 4.2.1 (21). For the outcome of whether or not a patient had a serum lactate measurement on day 1, we fitted a Targeted Maximum Likelihood Estimation (TMLE) model (22). From the TMLE model, we extracted and utilized the odds ratio (OR) to estimate the odds of receiving a serum lactate measurement. For the outcome of the number of serum lactate measurements during an ICU stay, we fitted a non-penalized, negative binomial regression [*statsmodel* package (23)] adjusted for confounders to estimate the number of serum lactate measurements for each patient each day in the ICU. We report our findings as incident rate ratios (IRR). All findings are reported with 95% CI and with White patients as the reference group.

## Results

### Baseline study cohort

**Figure 1:**
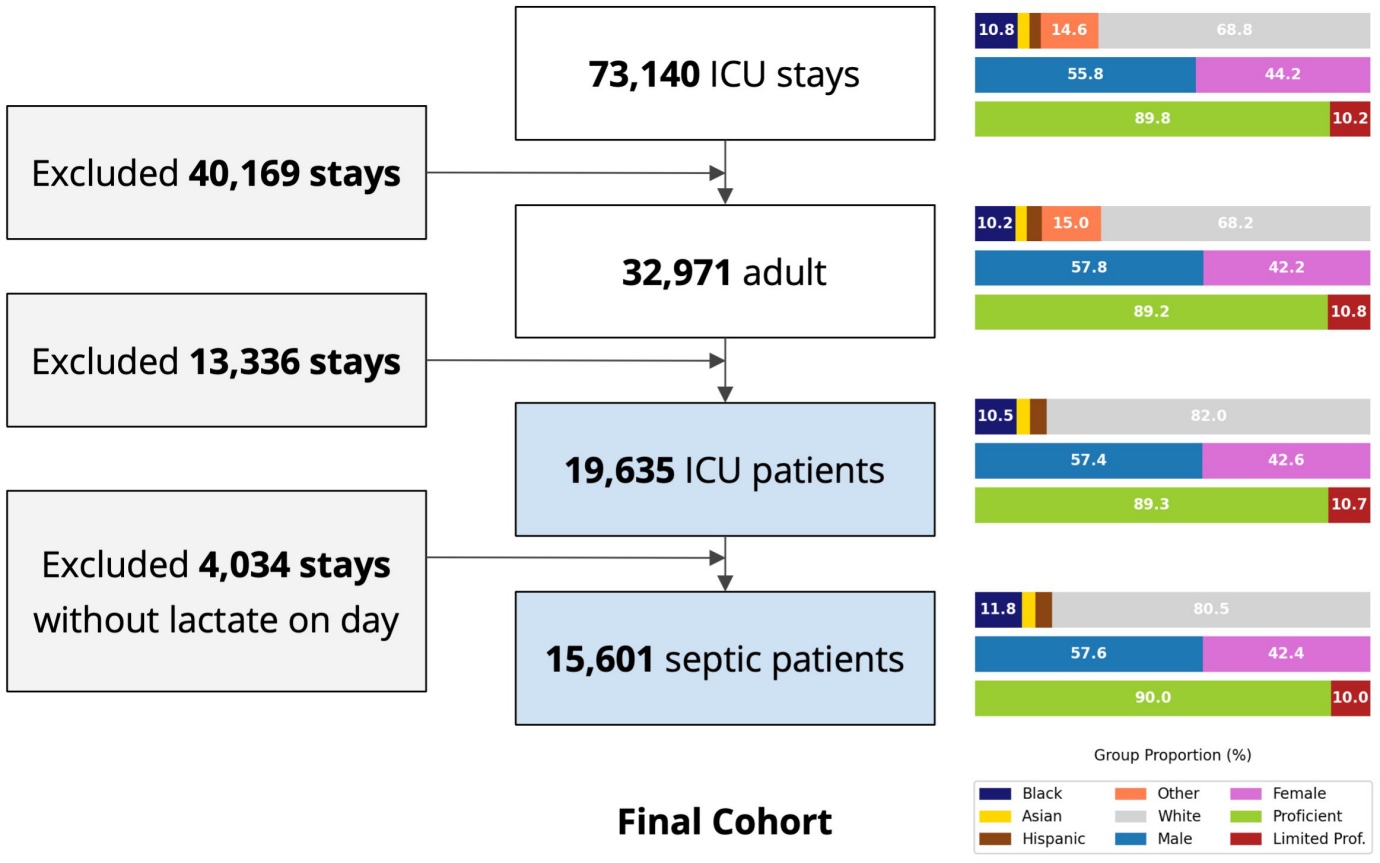
Study cohort selection flow chart, MIMIC-IV. Right panels depict the change of key demographic factors through application of the exclusion criteria. ICU, intensive care unit; LOS, length of stay; Proficient, English proficient; Limited Prof., limited English proficiency.

The MIMIC-IV database has 73,140 ICU stays, of which 15,601 were included in our final cohort following application of the inclusion and exclusion criteria (Figure 1). The race-ethnicity distribution was 10.8% Black, 3.8% Hispanic, 2.9% Asian, 68.8% White and 14.6% others (without specified race). The demographic distribution did not change after applying exclusion criteria.

SOFA score had a median of 6.00 (interquartile range (IQR) 4.00, 8.00; Table 1), regardless of the race-ethnicity reported at baseline, with the Charlson comorbidity index at 6.00 (IQR 4.00, 8.00). Serum lactate on day 1 was slightly higher in the Non-White group at 2.50mmol/L (IQR 1.60, 4.00), compared to the White group at 2.20 (1.50, 3.50). In addition, Non-White patients received more fluids on the first day in the ICU than White patients [2,060 ml (IQR 640, 5,000) vs. 1,690 (461, 4,540)], respectively. Of note, the volume of fluids received prior to admission to the ICU is not available in the dataset. Upon a reviewer's request, we added a supplementary breakdown of baseline characteristics and illness severity by race-ethnicity (White, Black, and non-White/non-Black) to enhance transparency regarding racial representation in the dataset (Supplementary Table S2).

**Table 1:** Baseline information on the study cohort, derived from MIMIC-IV.

	**Race and ethnicity**			
**Variables**	**Missing**	**Overall**	**Non-White**	**White**
*N* (%)		15,601 (100)	2,801 (17.9)	12,800 (82.1)
Age, median [Q1, Q3]	0	68.0 (57.0, 78.0)	64.0 (52.0, 76.0)	68.0 (59.0, 79.0)
Female sex, *n* (%)	0	6,520 (41.8%)	1,341 (47.9%)	5,179 (40.5%)
English proficient, *n* (%)	0	14,113 (90.5%)	1,894 (67.6%)	12,219 (95.5%)
Insurance Medicaid, *n* (%)	0	1,042 (6.7%)	398 (14.2%)	644 (5.0%)
Insurance Medicare, *n* (%)	0	7,476 (47.9%)	1,064 (38.0%)	6,412 (50.1%)
Insurance Other, *n* (%)	0	7,083 (45.4%)	1,339 (47.8%)	5,744 (44.9%)
Charlson comorbidity index, median [Q1, Q3]	0	6.00 (4.00, 8.00)	6.00 (4.00, 8.00)	6.00 (4.00, 8.00)
SOFA, median [Q1, Q3]	0	6.00 (4.00, 8.00)	6.00 (4.00, 9.00)	6.00 (4.00, 8.00)
Elective admission, *n* (%)		2,876 (18.4%)	312 (11.1%)	2,564 (20.0%)
Length of stay, days, median [Q1, Q3]	0	3.13 (1.83, 6.25)	3.21 (1.88, 6.83)	3.13 (1.83, 6.17)
Lactate day 1 (mmol/L), median [Q1, Q3]	0	2.20 (1.50, 3.50)	2.50 (1.60, 4.00)	2.20 (1.50, 3.40)
Number of lactate measurements day 1, median [Q1, Q3]		3.00 (2.00, 5.00)	3.00 (2.00, 5.00)	3.00 (2.00, 5.00)
Lactate day 2 (mmol/L), median [Q1, Q3]	9,397 (60.2%)	1.70 (1.20, 2.60)	1.80 (1.30, 2.90)	1.70 (1.20, 2.60)
Number of lactate measurements day 2, median [Q1, Q3]	9,397 (60.2%)	2.00 (1.00, 3.00)	2.00 (1.00, 3.00)	2.00 (1.00, 3.00)
Mechanical ventilation, *n* (%)	0	8,841 (56.7%)	1,566 (55.9%)	7,275 (56.8%)
Renal replacement therapy, *n* (%)	0	1,550 (9.9%)	397 (14.2%)	1,153 (9.0%)
Vasopressor(s), *n* (%)	0	9,243 (59.2%)	1,455 (51.9%)	7,788 (60.8%)
Fluids received day 1 (ml), median [Q1, Q3]	446 (2.9%)	1,750 (498, 4,620)	2,060 (640, 5,000)	1,690 (461, 4,540)

Q1, lower quartile range; Q3, upper quartile range; SOFA, sequential organ failure assessment.

### Model results

**Figure 2:**
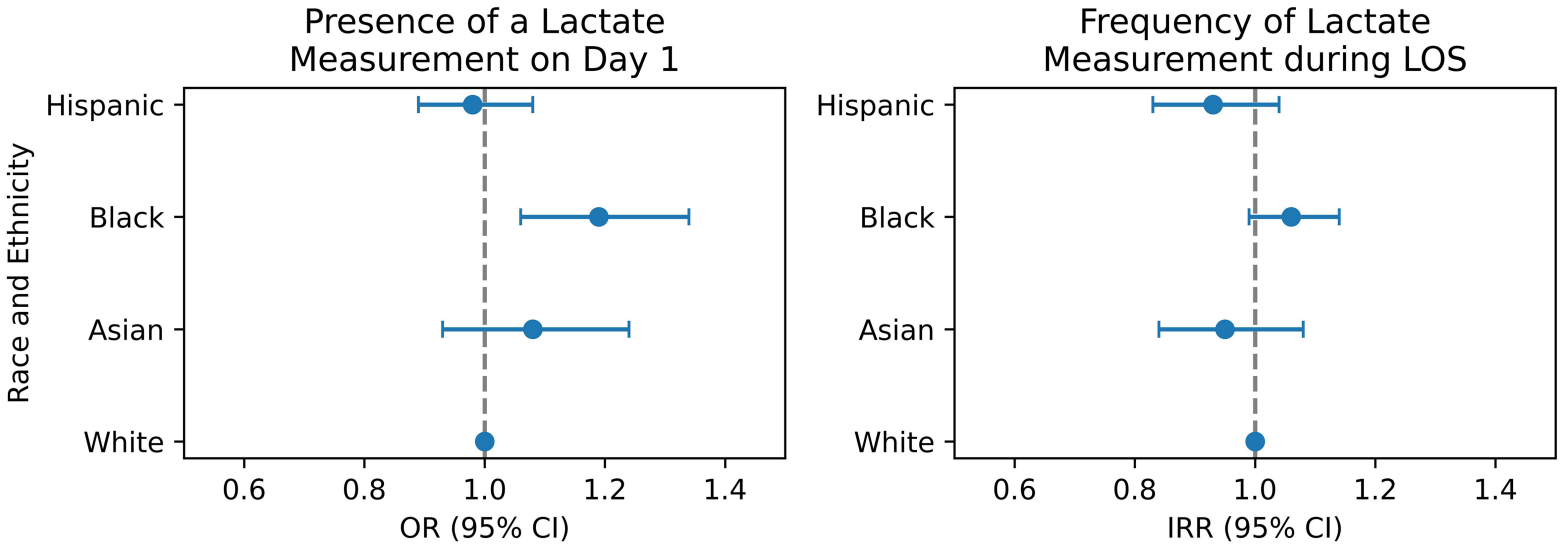
Summary of main results from TMLE **(left panel)** and negative binomial regression **(right panel)**. OR, odds ratio; IRR, incidence rate ratio; CI, confidence interval.

We adjusted our models for confounders according to a DAG (Supplementary Figure S1, Supplementary Table S1). Using the TMLE model with being White, male and English proficient as a reference, Black patients were more likely to have a serum lactate measurement on day 1 with OR 1.19 (95% CI 1.06, 1.34). Asian and Hispanic patients had a similar likelihood compared to White patients, with an OR of 1.08 (95% CI 0.93, 1.24), and an of OR 0.98 (95% CI 0.89, 1.08), respectively (Table 2, Figure 2a). We validated these findings with a cross-validated logistic regression model (Supplementary Table S3).

**Table 2:** Likelihood of receiving a lactate measurement on day 1 fitted by a targeted maximum likelihood estimation (TMLE) model.

**Demographic**	**OR**	**2.50% CI**	**97.5% CI**
White	Reference		
Black	1.19	1.06	1.34
Asian	1.08	0.93	1.24
Hispanic	0.98	0.89	1.08
Male	Reference		
Female	1.02	0.96	1.09
English proficient	Reference		
English non-proficient	0.96	0.86	1.07

OR, odds ratio; CI, confidence interval.

The negative binomial model was fitted to predict the total frequency of serum lactate measurements during a patient's ICU stay (Table 3, Figure 2b). We found no significant difference in the frequency of measurements across race-ethnicities compared to white patients as reference. Hispanic (IRR 1.12, 95% CI 0.99, 1.26), Black (IRR 1.01, 95% CI 0.94, 1.09), and Asian (IRR 1.08, 95% CI 0.95, 1.23) patients had a non-significant difference in their frequency of serum lactate measurements. In addition, English proficiency had no significant impact on measurement frequency (IRR 1.06, 95% CI 0.97, 1.16). On the other hand, female sex (IRR 0.94, 95% CI 0.90, 0.98) and having a urinary tract infection (IRR 0.68, 95% CI 0.50, 0.93) were associated with a decreased serum lactate measurement frequency, while having private insurance (IRR 1.07, 95% CI 1.02, 1.12) and being admitted electively (IRR 1.7, 95% CI 1.61, 1.81) significantly increased the frequency of receiving a measurement. Further, we conducted additional stratified analyses by admission SOFA score [cutoff at median of 6 supported by Ke et al. (24)] to explore differences in lactate measurement frequency by race and gender within illness severity strata (Supplementary Tables S4, S5).

**Table 3:** Results of the negative binomial regression for outcome of lactate measurement frequency on day 1.

**Variable**	**IRR**	**2.5% CI**	**97.5% CI**
Intercept	0.72	0.62	0.85
Age	1.00	1.00	1.00
Charlson comorbidity index	1.01	1.00	1.02
SOFA	1.10	1.09	1.10
Volume of fluids normalized by LOS	1.00	1.00	1.00
**Race**			
White	Reference		
Asian	1.08	0.95	1.23
Black	1.01	0.94	1.09
Hispanic	1.12	0.99	1.26
**Binary variables**			
Female sex	0.94	0.90	0.98
English proficient	1.06	0.97	1.16
Private insurance	1.07	1.02	1.12
Elective admission	1.70	1.61	1.81
Pneumonia	1.01	0.90	1.13
Urinary tract infection	0.68	0.50	0.93
Biliary infection	1.22	0.81	1.84
Skin infection	1.03	0.61	1.72

IRR, incidence rate ratio; CI, confidence interval; SOFA, sequential organ failure assessment; LOS, length of stay.

## Discussion

In this retrospective cohort study in patients with sepsis, we observed no discernible disparities between sexes and non-native English speakers in receiving a serum lactate measurement on day 1, although Black patients had a slightly increased likelihood. Furthermore, no apparent racial or language disparities were evident when examining the frequency of subsequent measurements, although a lower frequency was observed for women, those with private insurance, and those admitted electively. As Non-white patients were more likely to have Medicaid, there might still be disparities in care not captured in our data.

Although our study does not directly involve AI model development, its findings are highly relevant to the growing use of clinical data in artificial intelligence applications. Variability in measurement frequency, such as with lactate, can introduce biases into model training and deployment, particularly if the data reflects healthcare process differences rather than true physiological states. This is especially important given recent concerns about fairness and generalizability in AI models, which often underperform in underrepresented patient populations due to uneven data quality and representation (25, 26). Understanding and quantifying these real-world data characteristics is therefore, essential for building equitable and reliable AI systems in critical care. Health equity has become a priority in clinical research and among policymakers not only in the US but globally (27–29). In recent years, significant legislative changes around AI and health equity outcomes have been proposed and implemented. The European Parliamentary Research Service conducted a study on AI in healthcare in 2022 and recommended the implementation of specific coordination and support programs to address issues pertaining to AI and bias (30). In December 2023, the European Union approved the world's first legislation to regulate AI (31).

Beyond the obvious risks associated with feeding non-representative data to a model, variation in the clinical monitoring of patients presents a problem in the development of prediction, classification and optimization models using real-world data. The non-random sparsity of data from minoritized groups, even when represented in the dataset, has implications in the application of any statistical model. Machine learning-based decision support tools are an especially delicate area due to the sensitive nature of clinical decisions. Providers often intentionally refrain from measuring a variable especially in the ICU because of increasing recognition of the harm from over-testing (32). But the rationale behind such decisions is typically more complex, and confounded by both clinical and non-clinical (i.e., social determinants of care) features. In result, AI models learn wrong associations between clinical features and outcomes of interest. The problem becomes more pronounced in the advent of multi-modal modeling that requires black box deep learning representations (9). Models built on real-world data are thus subject to the human biases of the people who collected the primary data. For instance, a recent study found that large language models recommended low paying jobs more frequently to Mexicans, or implied that administrative work is solely a female job (33).

While our study did not assess mortality outcomes directly, partly due to the publication of similar work (34), the relationship between measurement frequency and patient outcomes remains an important area for future research. Causal inference methods, such as target trial emulation (35), could help determine whether more frequent monitoring leads to improved outcomes. In an effort to mitigate biases, some studies have suggested the use of causal inference frameworks for machine learning (33, 36, 37), which should help understand and avoid embedding biases into AI algorithms. Evaluating data inputs used in AI models for biases and disparities as done in our work is a prerequisite even before employing causal inference frameworks and should become standard practice as the understanding gained aids in building better, more equitable, and trustworthy AI models. This study provides a framework and approach for future work, as health care professionals, engineers, and developers have the moral accountability to ensure safe deployment of AI models (38, 39).

Lactate is frequently measured as part of bundled panels, such as point-of-care arterial blood gas analyses leading to synchronous measurement with other parameters. However, it can also be assessed independently in the central laboratory. In a previous study on blood glucose monitoring in the ICU, we compared point-of-care and central lab measurements and found no clinically relevant differences between the two methods (40), suggesting that the impact of measurement context may be limited in this setting. While our analysis was conducted using data from the MIMIC database, similar findings have been reported in other critical care datasets, including eICU and Duke. For instance, Matos et al. (41) observed patterns of variability in arterial blood gas measurements across these cohorts. This cross-dataset consistency suggests that our conclusions may be generalizable to other ICU populations and settings. The optimal frequency of monitoring of serum lactate measurement is unknown. Several recent studies and reviews have shown that serial lactate measurements and trends—such as peak levels, area under the curve, and clearance are associated with mortality in sepsis. For example, a 2023 nationwide Korean cohort study found that combining serial lactate values with SOFA improved mortality prediction compared to SOFA alone (42). A meta-analysis with roughly 4,400 patients suggests that a protocol focusing on lactate clearance leads to lower in-hospital mortality compared to ScvO2 normalization or usual care (43). A 2024 study in septic shock patients also linked initial, peak, and final 24-h lactate levels to 28-day mortality, although 24-h clearance was not predictive and optimal measurement frequency remained unclear (44). Similarly, a 2022 study using MIMIC-IV reported that lactate peak and AUC were associated with mortality, but lactate clearance was not more predictive than single values and performed worse than SOFA or NEWS scores (34). The current Surviving Sepsis Campaign guidelines support serial lactate monitoring but acknowledge that evidence for improving patient-centered outcomes remains limited and inconsistent (13). Many EHRs have already incorporated automated sepsis alerts to clinicians which rely on data such as the lactate to be present; disparities in collecting the data leads to disparities in usage of such alerts (45, 46). As such, the inputted data must be evaluated for bias. Other studies have shown that racially diverse Non-White ICU patients have nearly double the incidence of sepsis and higher rates of sepsis-related mortality compared to White patients (45, 47, 48). Furthermore, some studies in pediatric patients have reported higher mortality rates for those of lower socioeconomic status in the ICU (49). As such, all possible efforts need to be undertaken to close this disparity in patient care.

## Limitations

While our research provides valuable insights into the discourse on disparities and biases within critical care, it is essential to acknowledge the limitations of our study. Firstly, selection bias could be a potential concern, as our data only encompassed patients admitted to the ICU in an academic tertiary care center in the USA whose patients are predominantly White. However, data from MIMIC is generally very similar to data from eICU-CRD another publicly available database encompassing 208 ICUs in the US (50). In general, race-ethnicity is self-reported in MIMIC-IV or provided by relatives, however in instances where this was not possible, data is recorded by the providers themselves. Additionally, our study design precludes us from testing for unmeasured confounding variables, especially leading to confounding by indication. Future research endeavors should make concerted efforts to address these limitations, such as including Social Determinants of Health and fostering a more comprehensive understanding of the topic by employing causal inference frameworks as the next prerequisite step before validating AI models. Although our observed variability in lactate measurements was statistically significant, the absolute magnitude was small, mostly due to the large sample size, and may not be clinically meaningful. However, even small differences can carry relevance in the critical care setting, where clinical decisions often hinge on marginal changes. Recent studies have highlighted the challenges of bias and data completeness in electronic health records, particularly in underrepresented populations, which can impact both model performance and clinical interpretation (25, 26, 51). Our analysis focused on lactate due to its clinical relevance in sepsis and its frequent measurement in routine care, which allowed for robust assessment of intra-patient variability, but we agree that we cannot exclude incidental findings due to random variations. Also, similar disparities may affect other laboratory parameters (41), and our findings may not be generalizable to those. Future studies should explore a broader range of biomarkers to assess the extent of this issue across different clinical contexts. Moreover, future studies should extend their scope to cover other facets of care, including emergency departments, regular wards, or ambulatory care, to provide a more holistic perspective.

## Conclusion

The implications of our study extend beyond the realm of lactate monitoring during sepsis management. In addition to the ongoing challenge of achieving healthcare equity within a system marked by systemic biases, clinicians and researchers must remain cognizant of these disparities before endeavoring to enhance patient care at their local institution or constructing any AI model. These biases not only have the potential to distort predictions, but may also endanger patient's safety when the predictions are employed for treatment or management decisions.

## Data Availability

The data that support the findings of this study are available in MIMIC-IV with the identifier doi.org/10.1093/jamia/ocx084 publicly available on PhysioNet (https://physionet.org). The code that produces the results in this manuscript can be accessed at https://github.com/joamats/mit-lactate, which includes detailed instructions for running the code.
