# Supplementary material for: Potential source of bias in AI models: lactate measurement in the ICU in sepsis patients as a template
Source: Front Med (Lausanne). 2025 Jul 9;12:1606254. doi: 10.3389/fmed.2025.1606254 (PMC12283994; doi:10.3389/fmed.2025.1606254)
Supplement: Supplementary file 1 [file Data_Sheet_1.docx]

## **SUPPLEMENTARY MATERIALS**

### **Supplementary Figure 1. Directed acyclic graph for outcome of a single lactate measurement on day 1 of hospital admission**

**
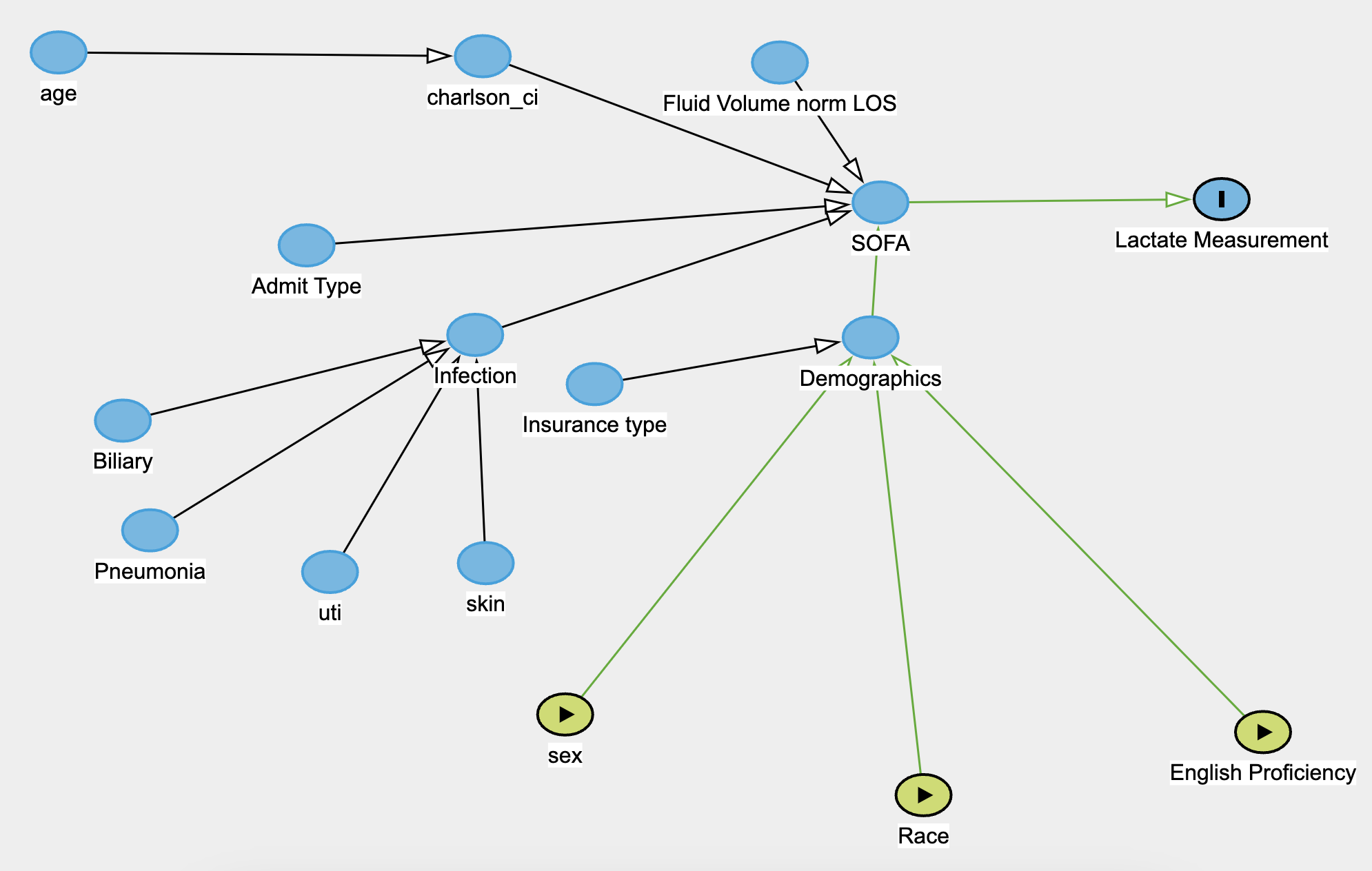
**

**Legend:** Race ethnic group includes Whites, Blacks, Hispanics, and Asians.

**Abbreviations:** charlson_ci, Charlson comorbidity score; SOFA, sequential organ failure assessment; uti, urinary tract infection; LOS, length of stay; REF, reference

### **Supplementary Table 1. Definition of considered covariates**

| **Covariates** | **Description** | **Handling of missing values** |
| --- | --- | --- |
| Sex | As provided by dataset | N/A |
| Age | At admission | N/A |
| Race Ethnicity | Grouped into Asian, Black, White, Hispanic, and Other | Exclusion of group “Other” |
| Insurance | As provided by dataset, Medicare/Medicaid or other | N/A |
| English Proficiency | As provided by dataset, English or limited | N/A |
| Year Group | As provided by dataset, bi-yearly bins | N/A |
| Elective Admission | As provided by dataset, true or false if admission is elective | N/A |
| Charlson comorbidity Index | As provided by dataset | N/A |
| Fluid Volume Normalized by Length of Stay | As provided by dataset | Mean Imputation |
| SOFA | SOFA score with each of its  subcomponents: on admission and for the selected 24 hours, aggregated by the maximum value | Assumption of best possible value in case of missing |
| Source of infection | ICD-10 codes J09.X, J1.X, J85.X, J86.X, N300.X, N390.X, K81.X, K830.X, K851.X, L0.X | Zero Imputation |
| Lactate | MIMIC-IV Item IDs 50813, 52442, 53154 | Assume no lactate measurement if no entries. |

**Legend:** X denotes a wild card character

**Abbreviations:** SOFA, sequential organ failure assessment

### **Supplementary Table 2. Baseline information on the study cohort stratified by White, Black, and non-White/non-Black race-ethnicity, derived from MIMIC-IV**

|  | Asian (N=527) | Black (N=1625) | Hispanic (N=649) | White (N=12800) | Overall (N=15601) |
| --- | --- | --- | --- | --- | --- |
| **Age overall (years),** Median (Q1, Q3) | 66.0 (54.0, 78.0) | 65.0 (54.0, 76.0) | 59.0 (47.0, 71.0) | 68.0 (59.0, 79.0) | 68.0 (57.0, 78.0) |
| **Sex,** Female | 216 (41.0%) | 865 (53.2%) | 260 (40.1%) | 5,179 (40.5%) | 6,520 (41.8%) |
| Female |  |  |  |  |  |
| **English proficiency,** Yes | 205 (38.9%) | 1,439 (88.6%) | 250 (38.5%) | 12,219 (95.5%) | 14,113 (90.5%) |
| **Insurance** |  |  |  |  |  |
| Medicare | 152 (28.8%) | 700 (43.1%) | 212 (32.7%) | 6,412 (50.1%) | 7,476 (47.9%) |
| Medicaid | 92 (17.5%) | 177 (10.9%) | 129 (19.9%) | 644 (5.0%) | 1,042 (6.7%) |
| Other | 283 (53.7%) | 748 (46.0%) | 308 (47.5%) | 5,744 (44.9%) | 7,083 (45.4%) |
| **Charlson Comorbidity Index (CCI),** Median (Q1, Q3) | 6.00 (4.00, 8.00) | 6.00 (4.00, 9.00) | 5.00 (3.00, 8.00) | 6.00 (4.00, 8.00) | 6.00 (4.00, 8.00) |
| **SOFA,** Median (Q1, Q3) | 6.00 (4.00, 9.00) | 6.00 (4.00, 9.00) | 6.00 (4.00, 8.00) | 6.00 (4.00, 8.00) | 6.00 (4.00, 8.00) |
| **Elective admission** | 83 (15.7%) | 136 (8.4%) | 93 (14.3%) | 2,564 (20.0%) | 2,876 (18.4%) |
| **Length of stay (days),** Median (Q1, Q3) | 3.04 (1.75, 6.11) | 3.38 (1.96, 7.13) | 3.08 (1.83, 6.58) | 3.13 (1.83, 6.17) | 3.13 (1.83, 6.25) |
| **Lactate day 1 (mmol/L),** Median (Q1, Q3) | 2.70 (1.70, 4.30) | 2.30 (1.50, 3.90) | 2.70 (1.70, 4.10) | 2.20 (1.50, 3.40) | 2.20 (1.50, 3.50) |
| **Number of lactate measurements day 1,** Median (Q1, Q3) | 3.00 (1.00, 5.00) | 3.00 (1.00, 5.00) | 3.00 (2.00, 5.00) | 3.00 (2.00, 5.00) | 3.00 (2.00, 5.00) |
| **Lactate day 2 (mmol/L),** Median (Q1, Q3) | 1.80 (1.38, 2.90) | 1.80 (1.20, 2.90) | 1.90 (1.30, 3.00) | 1.70 (1.20, 2.60) | 1.70 (1.20, 2.60) |
| Missing | 311 (59.0%) | 906 (55.8%) | 376 (57.9%) | 7804 (61.0%) | 9397 (60.2%) |
| **Number of lactate measurements day 2,** Median (Q1, Q3) | 2.00 (1.00, 3.00) | 2.00 (1.00, 3.00) | 2.00 (1.00, 4.00) | 2.00 (1.00, 3.00) | 2.00 (1.00, 3.00) |
| Missing | 311 (59.0%) | 906 (55.8%) | 376 (57.9%) | 7804 (61.0%) | 9397 (60.2%) |
| **Mechanical Ventilation** | 294 (55.8%) | 899 (55.3%) | 373 (57.5%) | 7,275 (56.8%) | 8,841 (56.7%) |
| **Renal Replacement Therapy** | 45 (8.5%) | 274 (16.9%) | 78 (12.0%) | 1,153 (9.0%) | 1,550 (9.9%) |
| **Vasopressor(s)** | 315 (59.8%) | 796 (49.0%) | 344 (53.0%) | 7,788 (60.8%) | 9,243 (59.2%) |
| **Volume of Fluids received (day 1) (mL),** Median (Q1, Q3) | 7760 (4790, 12500) | 7690 (3950, 13900) | 8100 (4790, 14300) | 7430 (4490, 13400) | 7500 (4470, 13500) |
| Missing | 1 (0.2%) | 3 (0.2%) | 0 (0%) | 33 (0.3%) | 37 (0.2%) |

**Abbreviations:** Q1, lower quartile range; Q3, upper quartile range; SOFA, sequential organ failure assessment

**Supplementary Table 3. Likelihood of receiving a lactate measurement on day 1 fitted by a logistic regression model**

| **Demographic** | **OR** | **2.50% CI** | **97.5% CI** |
| --- | --- | --- | --- |
| *White* | *Reference* | | |
| Black | 1.11 | 1.09 | 1.13 |
| Asian | 0.95 | 0.89 | 0.99 |
| Hispanic | 0.9 | 0.84 | 0.97 |
| *Male Sex* | *Reference* | | |
| Female Sex | 0.99 | 0.97 | 0.99 |
| *English proficient* | *Reference* | | |
| English non-proficient | 1.00 | 1.00 | 1.00 |

**Abbreviations:** OR, odds ratio; CI, confidence interval

**Supplementary Table 4a. Likelihood of receiving a lactate measurement on day 1 fitted by a Targeted Maximum Likelihood Estimation (TMLE) model for those with an admission SOFA score below the median of 6**

| **Demographic** | **OR** | **2.50% CI** | **97.5% CI** |
| --- | --- | --- | --- |
| *White* | *Reference* | | |
| Black | 1.13 | 0.99 | 1.29 |
| Asian | 1.12 | 0.96 | 1.31 |
| Hispanic | 1.01 | 0.87 | 1.17 |
| *Male* | *Reference* | | |
| Female | 0.98 | 0.90 | 1.06 |
| *English Proficient* | *Reference* | | |
| English Non-Proficient | 0.93 | 0.81 | 1.07 |

**Supplementary Table 4b. Likelihood of receiving a lactate measurement on day 1 fitted by a Targeted Maximum Likelihood Estimation (TMLE) model for those with an admission SOFA score at or above the median of 6**

| **Demographic** | **OR** | **2.50% CI** | **97.5% CI** |
| --- | --- | --- | --- |
| *White* | *Reference* | | |
| Black | 1.21 | 0.97 | 1.49 |
| Asian | 0.87 | 0.68 | 1.11 |
| Hispanic | 0.73 | 0.61 | 0.88 |
| *Male* | *Reference* | | |
| Female | 0.99 | 0.87 | 1.13 |
| *English Proficient* | *Reference* | | |
| English Non-Proficient | 1.21 | 0.97 | 1.50 |

**Supplementary Table 5a. Results of the negative binomial regression for outcome of lactate measurement frequency on day 1 for those with an admission SOFA score below the median of 6**

| **Variable** | **IRR** | **2.5% CI** | **97.5% CI** |
| --- | --- | --- | --- |
| Intercept | 0.36 | 0.29 | 0.45 |
| Age | 1.00 | 1.00 | 1.00 |
| Charlson comorbidity index | 1.02 | 1.01 | 1.04 |
| SOFA | 1.1 | 1.08 | 1.13 |
| Volume of fluids normalized by LOS | 1.00 | 1.00 | 1.00 |
| **Race:** |  |  |  |
| *White* | *Reference* | | |
| Asian | 1.13 | 0.95 | 1.36 |
| Black | 1.07 | 0.97 | 1.19 |
| Hispanic | 1.11 | 0.94 | 1.30 |
| **Binary variables:** |  |  |  |
| Female sex | 0.96 | 0.90 | 1.02 |
| English proficient | 1.01 | 0.90 | 1.14 |
| Private insurance | 1.01 | 0.95 | 1.08 |
| Elective admission | 1.57 | 1.45 | 1.69 |
| Pneumonia | 1.19 | 1.02 | 1.39 |
| Urinary tract infection | 0.65 | 0.46 | 0.92 |
| Biliary infection | 1.07 | 0.62 | 1.83 |
| Skin infection | 1.02 | 0.59 | 1.77 |

**Supplementary Table 5b. Results of the negative binomial regression for outcome of lactate measurement frequency on day 1 for those with an admission SOFA score at or above the median of 6**

| **Variable** | **IRR** | **2.5% CI** | **97.5% CI** |
| --- | --- | --- | --- |
| Intercept | 0.53 | 0.42 | 0.66 |
| Age | 1.00 | 1.00 | 1.00 |
| Charlson comorbidity index | 1.03 | 1.02 | 1.04 |
| SOFA | 1.07 | 1.06 | 1.08 |
| Volume of fluids normalized by LOS | 1.00 | 1.00 | 1.00 |
| **Race:** |  |  |  |
| *White* | *Reference* | | |
| Asian | 1.08 | 0.91 | 1.28 |
| Black | 0.97 | 0.89 | 1.07 |
| Hispanic | 1.09 | 0.92 | 1.28 |
| **Binary variables:** |  |  |  |
| Female sex | 0.99 | 0.93 | 1.05 |
| English proficient | 1.09 | 0.97 | 1.23 |
| Private insurance | 1.08 | 1.01 | 1.15 |
| Elective admission | 1.32 | 1.21 | 1.44 |
| Pneumonia | 1.07 | 0.93 | 1.23 |
| Urinary tract infection | 0.97 | 0.58 | 1.62 |
| Biliary infection | 1.16 | 0.68 | 1.97 |
| Skin infection | 1.39 | 0.27 | 7.22 |
